# Consequential research of accountability testing: the case of the CET

**DOI:** 10.1186/s40468-022-00165-6

**Published:** 2022-07-11

**Authors:** Yan Jin

**Affiliations:** School of Foreign Languages, Shanghai Jiao Tong University, Dongchuan Road 800, Minhang District, Shanghai, 200240 China

**Keywords:** Accountability testing, Test validation, Test consequences, College English Test

## Abstract

This article examines the changes taken place over the decades after a test-based accountability system was in place and proposes an agenda for consequential research of the testing program. The article starts with a review of theories of test-based accountability (Supovitz, Journal of Educational Change 10:211–227, 2009) and a socially grounded, integrated approach to test validation (Chalhoub-Deville, Language Testing 33:453–472, 2016; Chalhoub-Deville and O’Sullivan, Validity: Theoretical development and integrated arguments, 2020). Following Supovitz’s frameworks of accountability testing, the case of the College English Test (CET), a national English language testing program in China, is analyzed to explain the nature of the consequences of accountability testing and show the motivational effects, alignment effects, the informational role and the symbolic values of the accountability testing program. The case analysis highlights the importance of investigating consequences of an accountability test at the aggregate and the system levels. Guided by Chalhoub-Deville and O’Sullivan’s (Validity: Theoretical development and integrated arguments, 2020) integrated validity framework, the article delineates the claims for the theory of action argument and the communication engagement argument for future investigation of the consequences of the CET.

## Introduction

Assessments are developed and used not only for measurement purposes, but also for various educational and social functions. Assessment designers, therefore, need to coordinate the multiple perspectives on assessment purposes and anticipate the potential consequences on the educational, social, cultural, economic, and political systems within which they function (Newton, 2007, 2017). Language tests are developed and used for high-stakes decisions such as admission, graduation, migration, and so on. Tests for these purposes serve not only individual or institutional needs but also the needs on a broader level, i.e., driving the reform of language education and bringing about changes in educational and social systems. In China, assessments are often taken as an official endorsement of educational policies and employed as accountability measures to promote quality improvement. When educational reform is planned, assessments are revised or even replaced by new ones. Lin (2015), former Vice Minister of the Ministry of Education, recently announced a government plan to upgrade the national foreign language assessment system. The aim of the plan, according to Lin, was to promote educational reform in the new era in response to the needs of the national development strategy and international collaboration and competition. In this case, large-scale English language testing has been used as part of the government’s reform agenda for English language education.

In a reform-driven assessment context, high-stakes accountability tests, with a mission in educational reforms, can have potentially profound consequences for individuals, institutions and the society. Supovitz (2009) explains that “(I)f a test is used to hold individuals or institutions responsible for their performance and has stakes attached to it, it constitutes what is commonly called a test-based accountability system” (p. 213). In the literature of language testing and assessment, there is plenty of evidence to show that high-stakes language tests can have strong washback effects on teaching and learning (e.g., Cheng et al., 2004). There is, however, a paucity of literature on the mechanism and the impact of language tests designed for accountability purposes. Chalhoub-Deville (2016) cautions that reform-based testing “entails system (e.g., educational, economic, and social) change claims”, which “reach well beyond score interpretation and use at the individual or group level” (p. 454). Therefore, there is an urgent need to understand the nature of the consequences of a reform-driven language test and validate the test’s consequences at the aggregate and the system levels. In this article, the case of the CET is analyzed to examine the reform-driven nature of the testing program and argue for the importance of incorporating consequential research into validity frameworks.

## Literature review

### Test-based accountability

Tests for accountability purposes caught the attention of educational researchers in the 1990s, when there was an escalation of high-stakes testing for educational accountability in the U.S. High-stakes accountability testing reached a climax in 2001 when the No Child Left Behind (NCLB) initiative was launched. During the revision of the 1999 version of *Standards for Educational and Psychological Testing*, four main content areas of focus were identified, one of which was “increased use of tests for accountability and education policy-setting” (AERA et al., 2014: viii). In the revised version, the chapters “Educational Testing and Assessment” and “Testing in Program Evaluation and Public Policy” were rewritten “to attend to the issues associated with the uses of tests for educational accountability purposes” (ibid.: 4). In the 2014 version of the Standards, an accountability system is defined as “a system that imposes student performance-based rewards or sanctions on institutions such as schools or school systems or on individuals such as teachers or mental health care providers” (ibid.: 215).

Supovitz (2009) reports the major trends and influences of test-based accountability on American educators from the mid-1990s through 2008, which signify that “testing has become a widely utilized instrument for educational reform in America” (p. 211). Educational accountability assessments “provide data on individual student achievement and, aggregated, on comparative performance of schools, districts, states and national educational outcomes” (Cumming & Dickson, 2013: 222). In other parts of the world, the value of a test-based accountability system is also recognized and utilized. An example of the National Assessment Program (NAP) in Australia is cited below to illustrate the function of such a system.

The NAP made an announcement recently on its official website that “COVID-19 has had no significant impact on students’ literacy and numeracy achievement at the national or state/territory level, according to the NAPLAN (NAP Literacy and Numeracy) 2021 summary information” (https://www.nap.edu.au/home). This is an accountability message, which has been drawn on the basis of the results of a national assessment program. On its website, the NAP is introduced as “the measure through which governments, education authorities, schools and the community can determine whether or not young Australians are meeting important educational outcomes”. The benefits of the program are “to help drive improvements in student outcomes and provide increased accountability for the community”. At the system level, the NAP results provide education ministers with information about the implementation of their policies and resourcing in priority curriculum areas. The assessment also fulfills an accountability function by enabling the public to develop an understanding of student achievement at the local and national levels and of how their schools are performing.

### Theories of accountability testing

When a test is used as a tool for educational accountability, it goes beyond the traditional role of measurement and feedback. The primary goal of accountability testing is to evaluate educational effectiveness and make informed decisions on ways to improve education. By so doing, the system can potentially effect changes and improvements in the areas deemed necessary by policymakers.

Since the boom days of educational accountability in the early 2000s, educational researchers, particularly those in the USA, have been very interested in the mechanism of change in a reform-driven accountability system. In such a system, “﻿examinees’ test scores are also being used to make inferences about other people and systems that interact with the examinee—specifically, teachers, administrators, schools, school districts, and teacher preparation programs” (Sireci & Soto, 2016: 149). A test-based accountability system, therefore, is not merely an information system; rather, it is a system with stakes attached to it: Schools can be sanctioned, to the point of being closed, if performance criteria are not satisfied (Bennett & Gitomer, 2009: 45). Chalhoub-Deville (2016) comments on the Race to the Top (RTTT) project in the USA: “reform-driven RTTT moves beyond the traditional interest in individual student scores… (such projects) include far-reaching goals, which mandate attention to aggregate and system performance” (p. 455).

Supovitz (2009) proposes a set of frameworks against which trends of test-based accountability can be examined (p. 213-215). In his view, there are four theories that can explain the growth and prevalence of high stakes testing for accountability. The predominant theory is the motivational theory, which holds that “test-based accountability can motivate improvement”. The theory is proposed on the assumption that “educators lack the motivation to improve, and that incentives will address this”. Incentives could take the form of rewards or sanctions in the context of high-stakes testing. Secondly, the theory of alignment suggests that test-based accountability can “play a major role in spurring the alignment of major components of the educational system”. A coherent system of standards, curriculum and assessments is expected to perform at higher efficiency than an educational system with misaligned components. Thirdly, the informational theory argues that accountability systems “carry important information that educators can use to guide improvement”. Test data can be collected, aggregated and analyzed to improve classroom teaching and learning and enable policymakers to make informed decisions about educational programs. Finally, the theory of symbolism is founded on the notion that an accountability system “signals important values to stakeholders”. The essence of symbolism is public answerability, that is, an educational system is accountable to the general public. High-stakes testing is “a way of symbolically validating the authority of the prevailing education system”.

### Accountability and validity

In their conceptualization of consequences within validity, Chalhoub-Deville and O’Sullivan (2020) explicitly state that testing, especially with reform-driven systems, “is policy-driven and is intended to effect fundamental and encompassing changes at the individual, group and societal levels” (p. 142). Understanding the mechanism of change is not the entire mission of accountability testing. In the field of educational testing, validity is the overarching priority of the profession. Smith and Fey (2000) argue that the cultures of accountability and validity are at odds. In their view, when tests are used to serve high-stakes accountability functions, professional testing standards are often compromised. The Standards (AERA et al., 2014) has addressed this issue by stipulating that “(T)est-based accountability systems include interpretations and assumptions that go beyond those for the interpretation of the test scores on which they are based; therefore, they require additional evidence to support their validity” (p. 206).

In language testing, Chalhoub-Deville (2016) is the first to point out that claims made by accountability testing present language testers with “challenges not attended to in current validity theories” (p. 454). What is lacking are “structures, which make explicit the interconnections among policy stipulations, testing capabilities, and those impacted—at the individual, group, and societal levels” (ibid.). A recent line of research has therefore involved the conceptualization of the consequences of accountability testing within the validity framework. Quoting Bennett et al. (2011), Chalhoub-Deville (2016) stresses that “in addition to evaluations of score meaning and score-based use, the intended impact of the assessment implementation and its evaluation must, at least in this (reform-driven K-12) context, play a major role in validating testing programs” (p. 462).

Applying the theory of action (TOA), Chalhoub-Deville (2016) proposes a socially grounded framework for conceptualizing consequences within validity. The framework suggests that validity claims about score interpretations and uses need to be investigated at multiple levels: the individual level, the aggregate level and the educational-social contexts of testing. A measurement argument deals with the individual and aggregate-level specifications of claims (e.g., DIF, rater bias); and a TOA argument is associated with system-level investigations (e.g., the effect of assessment on curriculum). The notion of zone of negotiated responsibility is proposed to denote that circumstances may arise whereby assessment developers and users need to confer about and address consequential research during the life span of an assessment program. The notion is particularly useful for accommodating unallocated responsibilities for consequential research when assessment stakes are very high.

The following section presents a case analysis of the development and use of the College English Test (CET) over the decades. Since Supovitz’s (2009) frameworks were proposed on the basis of a comprehensive review of the literature surrounding high-stakes accountability testing and proved useful for analyzing the trends of accountability testing in the USA, the case analysis adopts the set of frameworks to understand the mechanism of the reform-driven language testing program in China and make a case for engaging stakeholders in test validation that “incorporates a social role for consequences” (Chalhoub-Deville, 2016: 453).

## A case analysis

### The reform-driven nature of the CET

China is known for its testing-oriented educational environment: *Sit for the exam and fight for the rank* was and still is not only a manifestation of the nature of competitiveness in all aspects and levels of educational assessment in China but also one of the key strategies used by the Chinese government to manage resources and social mobility (Cheng & Curtis, 2010; Yu & Jin, 2016). In such a context, policy enactment through an external, large-scale exam makes for an acceptable or even attractive proposition.

In the late 1970s, China inaugurated an open-door policy, which has become the cornerstone of its effort to revive the economy. English language ability was considered as an important soft power of the country. To support the open-door policy, the State Council decided to resume the university entrance examination in 1978, after a suspension of over one decade during the “cultural revolution” movement. The National Matriculation English Test (NMET) became a required component of the entrance examination in 1983. Since then, high-school students have been highly motivated to learn English and achieve good performances on the NMET, which accounts for one third of the total score of the entrance examination, “the most critical one in the lives of the Chinese youth and parents who care a lot about their children’s future” (Muthanna & Sang, 2016). Upon graduation from high schools, students achieve an average proficiency level of A2 to lower B1 on the CEFR (Zou, 2016), or CSE4 to lower CSE5 on China’s Standards of English Language Ability (CSE) (Jiang, 2019; Zhou, 2020). To become an “independent” (B2) or “proficient” (C1) user of English (Council of Europe, 2001), students must continue their English language education in universities. In the mid-1980s, the National College English Teaching Syllabus (State Education Commission, 1985, 1986) was developed and promulgated, in which College English was specified as a mandatory program for English language education at the tertiary level. College English has since become a basic foundational course for all non-English major university students, i.e., students who do not major in “English Language and Literature”.

To promote the implementation of the national teaching syllabuses, the College English Test (CET) was created to measure the English language proficiency of tertiary-level learners. The testing program was initiated by academia and endorsed by the Higher Education Department of the Education Commission (now Ministry of Education) in the mid-1980s (Fan & Frost, 2021; Jin, 2010, 2019; Yang, 2003; Zheng & Cheng, 2008). The governmental endorsement of the test was mainly reflected in the national teaching syllabuses, which stipulated that College English was a compulsory course for non-English major students and the CET could be given to the students as an end-of-course examination at two levels, Band 4 (CET4) and Band 6 (CET6). CET4 was designed for those who had completed the foundation stage of College English learning, and CET6 for those who had completed English learning at the more advanced stage.

In short, the CET, designed as a curriculum-based, program exit test, was aimed at promoting the reform of English language education at the tertiary level in China. The reform-driven nature of the CET has turned it into an accountability instrument. The CET stakeholders include the educational policymaker (Ministry of Education, MOE), the test provider (National Education Examinations Authority, NEEA), the test developer (National College English Testing Committee, NCETC), technology companies, higher education institutions, English language teachers and learners, and other users. These groups of stakeholders work within a hierarchical and interconnected structure, fulfilling different roles in operating the test-based accountability system.

### Motivational effects

High-stakes testing policies are proposed on the assumption that rewards, punishments, and resultant pressures are effective motivators for learning. Among the various stakeholders of the CET, university students are the most directly and strongly affected by the testing program. Therefore, this analysis of motivational effects sets out to understand whether and how the implementation of the CET has motivated English language learners in Chinese higher education institutions.

Zheng (2010) examined Chinese university students’ motivation, anxiety, global awareness, and linguistic confidence, and their relation to students’ performances on the CET Band 4 (CET4). Through the analyses of student questionnaire data (*n* = 830) and student interview data (*n* = 12), the study revealed the complex interactions among the context-specific social, educational and psychological factors and students’ performances on the CET4. The CET was generally perceived as a necessary component of College English learning, but the lack of a compulsory speaking test had negatively influenced English learning. Students’ instrumental motivation, including mark orientation, further-education orientation, and job orientation, were prominent in affecting their learning English, which in turn influenced students’ achievement in the CET4 written test.

Adopting the self-determination theory (SDT) (Deci & Ryan, 2000; Ryan & Deci, 2000), Jin and Cheng (2013) and Cheng et al. (2014) investigated how motivation and test anxiety, the two primary cognitive factors, affect test takers’ performances on three high-stakes language tests: the Canadian Academic English Language (CAEL) Assessment in Canada, the CET in the Chinese mainland, and the General English Proficiency Test (GEPT) in Taiwan. The CET data were collected from 493 Chinese university students, including their CET scores and responses to surveys on test value, motivation and test anxiety. The results showed that the students had a very high evaluation of the importance of the CET and they reported a higher level of motivation for English learning than the CAEL and GEPT respondents, because college graduates entering the workforce are expected to have a CET certificate and top-tier cities grant residential permits to college graduates with a CET certificate (see also Jin, 2014). Learners’ perception of the test’s value had a significant effect on their learning motivation and test anxiety. The study confirmed that motivation and test anxiety had a significant predictive power of CET scores, accounting for 18% variance of the total score. It was also found that students reporting higher levels of intrinsic motivation achieved higher CET scores and that those with higher levels of external regulation obtained lower CET scores. It is suggested that intrinsic motivation could be influenced by test importance to self, whereas high-takes uses of the test may have led to stronger extrinsic motivation.

Using high-stakes tests to drive reform, however, could be a simplistic “carrot and stick approach” (Ryan & Brown, 2005; Ryan & Weinstein, 2009). In his framework of test-based accountability, Supovitz (2009) also draws attention to an important caveat to the motivational theory: extrinsic motivation may affect in a complex way teachers’ and leaners’ intrinsic motivation to improve. Therefore, the motivational implications of high-stakes test-based reforms need to be carefully explored and researched through empirical investigations.

### Alignment effects of the test-based accountability system

**Fig. 1:**
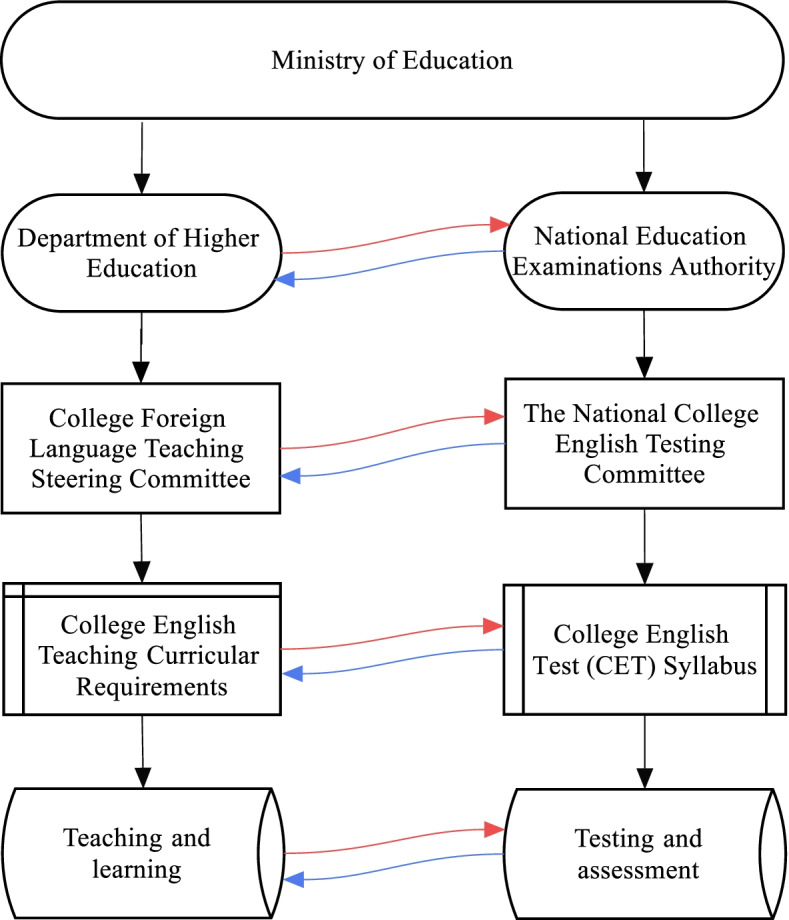
An interconnected system of College English education

A desirable function of an accountability test is to spur the alignment of major components of the educational system. In the College English program, curricular requirements provide essential guidance to program developers, material developers, instructors, learners, and assessors. As a key component of this curriculum-based accountability system, the CET has been closely aligned to the objectives of English teaching and learning at the tertiary level. Meanwhile, the implementation of the test has powerful washback on teaching and learning. Figure 1 illustrates the interconnected system of College English teaching, learning, and assessment.

In Chinese tertiary-level English language education, policymakers and implementers work within a hierarchical and interconnected system. Policies of foreign language education at the tertiary-level are made by the Higher Education Department (HED), which sets up the College Foreign Language Teaching Steering Committee. The current committee consists of 57 professors from different universities across the country. The committee is responsible for developing and promoting the implementation of curricular requirements. The assessment component of the system is managed by the NEEA, a government institution in charge of large-scale education examinations. Policies on the development and reform of the CET are implemented by the NCETC. The current committee consists of 25 professors and three senior advisers. Both the HED and NEEA are supervised by the Ministry of Education, which ensures a close connection between the teaching and assessment systems. There is also a large overlap between the teaching committee and the testing committee, providing an effective guarantee to the alignment between College English teaching requirements and assessment objectives.

Over the decades, there have been major revisions or minor changes to the CET so as to ensure that the assessment criteria of the test are closely aligned to the curriculum. In the earlier versions, much importance was attached to receptive skills, especially reading comprehension. Discrete-point items were used to assess learners’ knowledge of vocabulary and grammar. Text-based, multiple-choice cloze was used as an integrative task to assess learners’ linguistic knowledge. In the recent revision, the CET has employed new tasks to assess expeditious reading, Chinese to English paragraph translation, listening comprehension of lectures, and so on. To draw attention to speaking, the CET Committee launched the CET Spoken English Test (CET-SET) in 1999. In 2013, the computer-based CET-SET was rolled out to further promote the teaching and learning of English speaking.

Washback studies, unsurprisingly, have yielded mixed results. There is evidence of teachers’ positive perceptions of the CET reform and of positive impact of the CET on classroom teaching, for example, smaller class size, more practice of listening, fast reading, and translation, less training of test-wise strategies, and so on (e.g., Gu & Peng, 2010; Yang et al., 2013). According to Yang et al. (2013), teachers’ understanding of curricular requirements “relies mainly on textbooks and the CET test syllabus” (p. 320), which may explain the strong impact of CET reform on teaching and learning. However, there is also evidence that the CET reform had little impact on classroom teaching. Zhan (2010) explored the complicate mechanism of CET washback through focus group discussions and individual interviews with 15 instructors and 2 deans. The study found that the test designers’ intention of promoting positive washback by revising test content and format were constrained by multiple factors, including mainly teachers’ lack of assessment literacy, inadequate communication between the test developer and test users, and the high stakes attached to the test (e.g., as a requirement of college graduation).

It is worthwhile to point out that any element(s) in Fig. 1 can produce misalignment. It is the misalignment among the interconnected system that is likely to produce unintended washback and consequences within this socio-educational context in China. A case in point is that the test syllabus may become the de-facto curriculum, raising the issue of “the tail wagging the dog” (Li & Zeng, 2002). Over the decades, a recurring theme of the discussion on large-scale testing programs, also a major criticism of such programs, is the so-called teaching and learning to the test, typically characterized by narrowing the teaching content, replacing classroom teaching with test preparation, and stop teaching and learning after obtaining a certificate (e.g. Chapter 7 in Cai, 2006; Han et al., 2004). To achieve a more coherent language education system, the NEEA has recently developed the China’s Standards of English of English Language Ability (CSE) (Liu & Wu, 2020). The expectation is to use the language framework as a guide to bring curricula, pedagogy, and assessment into fruitful interaction with one another. Figueras (2012), however, questions the effectiveness of language frameworks for transforming classroom teaching and learning. The implementation of the CSE therefore may face challenges at both macro and micro-political levels (Jin et al., 2017).

### Information for guiding teaching and learning

Reporting scores to intended users is a critical part of large-scale testing and serves as the primary interface between the test developer and stakeholders (Roberts & Gotch, 2019). Standard 6.10 of the Standards for Educational and Psychological Testing (AERA et al., 2014) states that “(W)hen test score information is released, those responsible for testing programs should provide interpretations appropriate to the audience” (p. 119). It is also noted that the interpretations should “describe in simple language what the test covers, what scores represent, the precision/reliability of the scores, and how scores are intended to be used” (ibid.).

The CET written tests are “criterion-related and norm-referenced” (Yang & Jin, 2001). As mentioned above, the assessment criteria are aligned to College English curricular requirements, rendering the testing program criterion-related. The scores are reported in a norm-referenced system. A norm was established when the test was launched in the mid-1980s. Therefore, reported scores of the CET written tests carry two pieces of information: (1) to demonstrate whether a student’s performance has met the requirements specified in the curriculum, and (2) to show how well the student has performed compared to the norm group. The CET speaking test (CET-SET) is administered separately and the results are reported in a grade (A+, A, B+, B, C+, C, and D). Grade descriptions are provided on the score report.

**Fig. 2:**
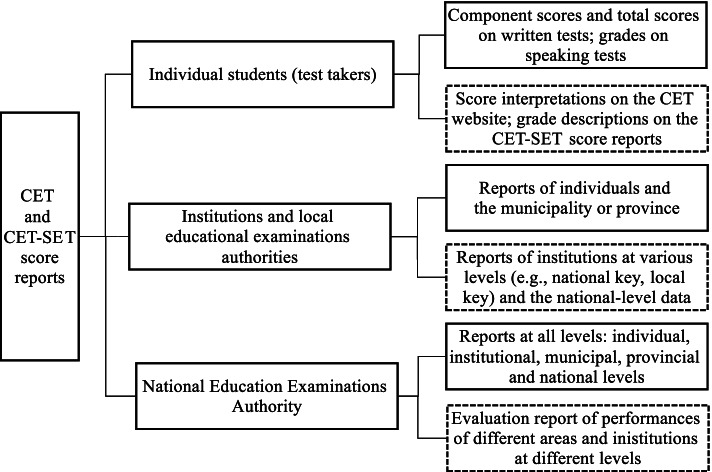
CET score reporting: recipients and contents

CET test results are reported at the individual and the aggregate levels (see Fig. 2). Component and total scores are reported to students, institutions, local educational examinations authorities, and the NEEA (see the boxes in solid lines on the right side of the figure). Score interpretations are provided in the test syllabus which is available on the test’s official website (http://cet.neea.edu.cn/), including an explanation of the norm-referenced score, examples of score interpretations, and percentile tables for each component score and the total scores. To provide students, institutions, and examination boards with broad frames of reference, the CET Committee also reports performances at the group and national levels (see the boxes in dotted lines on the right side of the figure). The committee provides an internal report upon request by the NEEA to describe performances of test takers from different areas of the country and institutions of different tiers and to analyze the discrepancies by incorporating survey data about contextual facets such as teaching hours, credits, learning materials, teachers’ beliefs and attitudes, institutional policies of score uses, and so on. The report has not been released for fear that some of the statistics might be misinterpreted, leading to unfair competition among institutions of different types or in different areas.

Information at the group level facilitates decision-making on teaching and learning. For example, a comparison of the CET scores during the first, second and third 5-year periods (from 1987 to 2002) shows that students’ performances on writing improves the most among the components of listening, reading and writing (Jin & Yang, 2006). Writing had been a compulsory component since the inception of the CET, but test takers, especially those with a low level of writing proficiency, often chose to give up writing and spend time on the reading section, in the hope of getting a higher score. In 1990, writing was made a separate component and the test time was strictly controlled, i.e., students were not allowed to work on other sections during the writing test. To further promote the teaching and learning of writing, a policy was implemented by penalizing those who did not work on the writing task: a zero score would fail the test taker on the whole test. Jin and Yang (2006) reported that over the 15 years, the national average of the reading score increased from 25 to 27.5 (out of 40), the listening score from 10 to 12.5 (out of 20), and the writing score from 4.5 to 7.5 (out of 15), indicating greater attention to English writing by teachers and learners.

Test performances could also be compared at group levels to understand the possible influence of social and educational contexts on learners’ English language proficiency. In the mid-1990s, the CET Committee used average graded scores (AGS, see Jin & Yang, 2006 for the meaning and calculation of AGS) to describe group performances. The example quoted in Jin and Yang (2006) shows discrepancies among different test taker groups: 159.2 for the whole test population, 203.5 for 89 national key universities, 147.4 for non-key universities; 187.5 for 7 provinces in coastal areas, 150.7 for 14 provinces in central areas, and 123.6 for 10 provinces in western areas. The results indicate a huge imbalance in English language proficiency among students in key and non-key universities and among students from economically more developed and less developed areas of the country.

Informative as they are, norm-referenced scores present a challenge to test users. To improve the meaningfulness of CET scores and the transparency of CET score interpretation, the committee recently conducted an alignment study to link the CET to the CEFR and the CSE (Jin et al., 2022). Reform has also been planned for CET score reporting so that the test results will carry more explicit criterion-related meanings and be reported at a more granular level.

### Symbolic values for stakeholders

Assessments are not only perceived as a strategy to promote educational reform but also deployed as evidence of the accountability of an educational system. Supovitz (2009) points out that a test-based accountability system carries symbolic values for its stakeholders. When the stakes of the CET are high, the values attached to the testing program increase, making it an increasingly powerful socio-political tool. A revealing example of the symbolic use of the CET is that in some private or low-ranking universities, CET certificates are required for graduation (Wang, 2011). The minimum score policy is implemented partly to show to the public that graduates of the institutions have achieved a certain level of English language proficiency. That is, the minimum score is construed as a sign of the quality of education, not simply a requirement of learners’ English proficiency.

The CET is also instrumental in upholding the status of College English as a basic foundational course and securing a minimum of 6 to 8 credits for English classes during the first 2 years in universities. In the Chinese higher education system, a fixed number of credits are given to so-called foundational courses. More credits to College English would mean a reduction of the credits in other foundational courses. Since students’ performances on the CET are taken as an indicator of the quality of college graduates, the College English program has been given sufficient attention by educational administrators in universities. Teachers may even receive bonus payments if their students perform well in the test. Though teachers complain about the stress caused by the anxiety to achieve a good score of their students, they also see the value of the test in increasing their job security and bringing them job satisfaction.

In addition, the CET has an added value of allowing students to access a locally developed language test without a heavy financial burden. Compared with international tests, the CET is a low-cost option, due largely to its large-scale and non-commercial nature. Currently, the registration fee of the written tests range from 4 USD to 6 USD depending on the location of the institution (1USD is about 6 to 7 RMB); and the fee of the speaking tests is about 8 USD. More importantly, students also save the time for preparing for an international test because local organizations or companies use CET certificates for recruiting college graduates.

## Discussion

### The need for consequential research of the CET

The case analysis indicates that educational policymakers recognize the value of large-scale assessments and make best use of them for education management and reform. However, in the field of language assessment, the impact of reform-based accountability testing on educational and social systems is often discussed but seldom empirically investigated. Studies of the washback on teaching and learning have been conducted and reported, but mostly at the individual or group levels. To hold language testing professionals accountable for test consequences, there is an urgent need for evidence-based validation which includes studies of consequences in the educational and social contexts.

Consequential research of reform-driven testing necessitates the conceptualization and investigation of test consequences at the aggregate or group level where tests are used by institutions for decision-making and at the system level, i.e., the educational-social contexts of testing. In Chalhoub-Deville and O’Sullivan’s (2020) socially grounded, integrated framework for conceptualizing consequences within validity, four types of arguments are specified for a comprehensive validation of language tests: the test development argument; the measurement argument; the theory of action argument; and the communication engagement argument. Of particular concern to consequential research of reform-driven accountability testing are the latter two types of claims, which are laid out below to guide future consequential research of the CET.

### Claims for the theory of action argument

To incorporate a social orientation to consequences in validity research, theory of action (TOA) is needed to outline the intended consequences for stakeholders and to mitigate unintended consequences (Sireci & Soto, 2016). Based on Kane’s (2006) notion of interpretative argument, Bennett et al. (2011) suggest a two-part interpretive argument consisting of measurement-argument and TOA components for validating an assessment program for accountability purposes. In their view, this type of program should not only “function well as assessments”, but also “function effectively as part of the educational programs in which they are embedded” (p. 3).

Chalhoub-Deville and O’Sullivan (2020) also suggest that TOA arguments be put in place at the outset of an accountability testing program to “indicate what exactly we would like to achieve with our testing programme, i.e., planned outcomes or consequences” and to “build into its plans investigations to address unintended practices, test score interpretations, decisions and uses” (p. 151–152). In Table 1, consequences in a TOA research plan are articulated for different stakeholders of the CET. The claims address the intended uses of the CET for promoting educational reform as well as its gate-keeping functions, which are not intended by the test developer.

**Table 1:** Claims for the TOA argument of CET consequences

**Education and assessment policymakers: MOE, NEEA**	
1. The CET is instrumental in promoting the reform of tertiary-level English language education.	
♦ Contributing to the improvement in the quality of tertiary-level English language education.	
♦ Contributing to the improvement in tertiary-level learners’ English language proficiency.	
♦ Strengthening the employability skills of college graduates.	
♦ Enhancing the college graduates’ ability to participate in international collaboration.	
2. The implementation of the CET helps to improve social justice and fairness.	
♦ Increasing learners’ opportunity for further education.	
♦ Ensuring learners’ access to English language assessment.	
♦ Opening up new opportunities and creating social mobility.	
3. The CET prevents English language education at the tertiary level in China from an over-reliance on international language tests.	
**Test developer and users: NCETC, tech companies, institutions, teachers and learners**	
4. The CET has positive washback on tertiary-level English language teaching and learning.	
♦ Increasing tertiary-level learners’ motivation for English learning.	
♦ Promoting the teaching-learning-assessment alignment of the College English program.	
♦ Providing useful information for improving College English teaching and learning.	
5. Test fairness is ensured in the development and administration of the CET.	
♦ Effective measures are adopted to prevent cheating.	
♦ Item bias is avoided through test review and DIF analysis.	
♦ Accommodation is provided to those in need.	
**Other users (e.g., employers, admission officers, local government officials)**	
6. The decisions based on the CET results are appropriate and justifiable.	

It should be noted that these claims are made in a very broad and general manner. They are not meant to be exhaustive and are expected to be broken down to sub-claims in future validation studies so as to provide useful guidance for empirical investigations. The most important claim is about the reform of tertiary-level English language education. Evidence needs to be collected to show whether the implementation of the CET has contributed to improving the quality of tertiary-level English language education, students’ English language proficiency, college graduates’ employability, and their ability to participate in international collaboration. In terms of social justice and fairness, attention needs to be paid to the impact of the testing program on students’ learning opportunities when test scores are used for graduate program admission, learners’ access to English language assessment when factors such as accessibility and cost are considered, and social mobility when test scores are used for decisions on residential permits. It is also worth investigating the role of the CET in preventing tertiary-level English language education from an over-reliance on international language tests.

Washback on teaching and learning is a key concern of the test developer, higher education institutions, teachers, and learners. As reported in the previous section, college students have a high extrinsic motivation for English learning due to the high-stakes uses of the CET. However, the relationship between the extrinsic and intrinsic motivation and their combined effect on teaching and learning need further exploration. Evidence is also needed for the alignment effects of the CET on the other elements in the educational system, such as curriculum, teaching practice, teachers’ beliefs, learner autonomy, and so on. The side-effect of the alignment, i.e., teaching/learning to the test, is also worth further exploration. With respect to the informational role of the test, the CET reports total and component scores, profiling test takers’ performances on different component skills (listening, reading, writing, and translation). The effectiveness of the score report, however, needs to be evaluated in future consequential studies.

Another major concern of the test provider and the test developer in recent years is high-tech cheating, which could seriously jeopardize test fairness. To combat cheating, the CET committee, under the supervision of the NEEA, has designed and implemented the practice of multiple versions (the same source texts and questions, but presented in different orders) and multiple forms (different source materials and different questions) in the CET written tests. That is, an item bank is used for each test so that test takers have no way to identify the particular version or form they are taking, making it impossible for them to cheat by using high-tech devices (Jin & Wu, 2017). Outcomes of the preventative measure need to be further investigated to demonstrate its value for improving test fairness.

The claim about test uses for various gate-keeping functions is probably the most difficult to validate in consequential research of reform-driven accountability tests, due to the challenge in the allocation of responsibilities. At the early stage of the CET development and operation, the test developer took up the responsibility of test validation. With the rising stakes and expansion in the uses of the CET, the situation becomes more complex and it is increasingly difficult to allocate the responsibilities for test consequences and the research of the consequences. Chalhoub-Deville (2016) suggests that “under such circumstances, stakeholders have a shared responsibility to discuss capabilities to fund or undertake research for consequences” (p. 468). It should therefore be stressed that for CET consequential research, we need to build the synergy of key players and engage all stakeholders in promoting beneficial consequences of the large-scale test.

### Claims for the communication engagement argument

The goal of communication engagement is to “render technical information meaningful to those who will need to work with that information and to turn it into usable actions” (Chalhoub-Deville & O’Sullivan, 2020: 154). During the test development stage, it is important to communicate design principles to item writers, the platform developer and psychometricians so as to “build consequences into the test design”; at the operational stage, it is essential to “provide meaningful, relevant and understandable information” to stakeholders to enable them to make informed decisions on test uses (ibid.: 153–154). In Table 2, claims for the communication argument are articulated to guide future consequential research of the CET.

**Table 2:** Claims for the communication argument of CET consequences

Stakeholder	Communication engagement claims
MOE	1. Educational policymakers are well informed of the benefits and pitfalls in using the test to promote educational reform.
NEEA	2. The test provider is engaged in an active communication with stakeholders, especially educational policymakers and other decision makers.
NCETC	3. The test developer is engaged in an active communication with stakeholders, especially technical support teams, teachers and learners.
Technology companies	4. Technical teams understand the design principles and requirements of the test and are able to meet the needs of test delivery and scoring.
Institutions	5. Higher education institutions understand CET scores and are able to make appropriate decisions on the uses of test scores.
Teachers	6. Teachers are equipped with an adequate knowledge about the test so as to better prepare learners for the test and make best use of the test to improve teaching and learning.
Learners	7. Learners are provided with sufficient information about the test so as to better prepare for the test and use test results to improve learning.
Other users	8. Users are equipped with an adequate knowledge about the test to make informed decisions on test uses for various gate-keeping functions.

The most challenging task for communication engagement is to create channels of communication and find appropriate ways to engage different stakeholders. Knoch (2021) analyzed three instances of policy advice to demonstrate the complexities involved in providing expert advice in policy contexts. In Lo Bianco’s (2021) term, experts are engaged in “policy conversations”, i.e., “a special kind of dialogue in which language testers are positioned as experts interacting with officials who are positioned as decision makers” (p. 87). The key issue, according to Lo Bianco, is about “how experts interact with powerholders” (p. 88). Communication experts, as suggested in Chalhoub-Deville and O’Sullivan (2020), may help, but communication with policymakers and test users should remain the primary responsibility of the test provider and the test developer, who are most knowledgeable about the testing program.

In the case of the CET, it is essential for educational policymakers, mainly the MOE, to be well informed of the benefits and pitfalls in using large-scale testing to promote educational reform. The main responsibility of communicating with educational policymakers may fall on the NEEA, the test provider. It is also important for the NEEA to communicate with decision makers who use the CET for various gate-keeping functions. For the CET Committee, effective communication with the technical support team, teachers and learners would help ensure the quality of its testing services so as to achieve so-called impact by design. Teachers are also a major stakeholder group in this communication plan. Equipped with a good level of assessment literacy and an adequate knowledge about the CET, College English teachers will be in a stronger position to make best use of the test to improve teaching and learning and prevent teaching or learning to the test.

## Conclusion

Large-scale and high-stakes testing is often used as a policy instrument to leverage changes in educational and social systems. Empirical validation in the field of language testing, however, has seldom been directed at the consequences of reform-driven testing. In this article, the case of the CET is analyzed to highlight the importance of “engaging in the real-world consequences of our testing programs” (Chalhoub-Deville, 2016: 467). The case analysis shows that Supovitz’s (2009) theories of accountability testing are useful for deepening our understanding of the mechanism of the test-based accountability system in the context of Chinese tertiary-level English language education. The analysis demonstrates that the CET has motivational effects on teaching and learning and facilitates curricular alignment. The test also produces useful information for decision making and has symbolic values for stakeholders. The system-level impact of the testing program on educational reform and social development, however, is rarely researched and documented in the literature.

In the foreword to an edited volume on assessing Chinese learners of English, Weir (2016) points out that “(G)iven the huge numbers of students whose lives are affected by local and international English language tests it is critical that test providers understand how policies and practices of assessing Chinese learners of English as a foreign language are intertwined with the social, political and educational systems in which the tests operate and in turn impact upon” (p. x). Supovitz (2009) suggests that the future direction of accountability testing is to explore “how test-based accountability systems can best be used to leverage meaningful improvements in the educational system” (p. 212). Following the case analysis, validity claims for the TOA argument and the communication engagement argument have been articulated to guide future consequential research of the CET. Given the nature and extent of consequential research, evidence gathering efforts should be ongoing, sustainable, and integrated into the routine operation of the testing program. Though the consensus is that all stakeholders should share the responsibility of consequential validation, test providers and developers have been entrusted with more responsibilities, which call for a commitment to an effective and efficient test-based accountability system to promote educational and social development.

## Data Availability

Not applicable.

## Abbreviations

CAEL: Canadian Academic English Language
CEFR: Common European Framework of Reference for Languages
CET: College English Test
CET-SET: College English Test—Spoken English Test
CSE: China’s Standards of English Language Ability
GEPT: General English Proficiency Test
HED: Higher Education Department
MOE: Ministry of Education
NAP: National Assessment Program
NAPLAN: National Assessment Program Literacy and Numeracy
NCETC: National College English Testing Committee
NCLB: No Child Left Behind
NEEA: National Education Examinations Authority
RTTT: Race to the Top
SDT: Self-Determination Theory
TOA: Theory of Action
